# Treatment of Dental Anxiety and Phobia—Diagnostic Criteria and Conceptual Model of Behavioural Treatment

**DOI:** 10.3390/dj9120153

**Published:** 2021-12-17

**Authors:** Ulla Wide, Magnus Hakeberg

**Affiliations:** 1 Department of Behavioral and Community Dentistry, Institute of Odontology, Sahlgrenska Academy, University of Gothenburg, 405 30 Gothenburg, Sweden; hakeberg@gu.se; 2 Public Dental Service, Region Västra Götaland, 402 33 Gothenburg, Sweden

**Keywords:** dental anxiety, dental phobia, adults, diagnosis, treatment manual, cognitive behaviour therapy, interdisciplinary collaboration, dental public health

## Abstract

Dental anxiety and dental phobia are still prevalent among adult individuals and should be considered a dental public health issue. Dental anxiety/phobia is often described as a vicious cycle where avoidance of dental care, poor oral health, and psychosocial effects are common features, often escalating over time. Treatment should include therapy for dental anxiety/phobia and oral diseases. This paper discusses aetiology, prevalence, and diagnosis of dental anxiety/phobia and, in detail, presents a conceptual treatment model at the Dental Fears Research and Treatment Center in Gothenburg, Sweden. In addition, based on systematic reviews, evidence-based treatment for dental anxiety is revealed including the interdisciplinary approach between psychology and dentistry.

## 1. Introduction

Dental anxiety and dental phobia are still prevalent among adult individuals and should be considered a dental public health issue. Dental anxiety/phobia is often described as a vicious cycle where avoidance of dental care, poor oral health, and psychosocial effects are common features, often escalating over time [1]. Thus, it has been of utmost interest in the research on dental anxiety to explore longitudinal data in order to analyse the complexity of dental anxiety/phobia. Moreover, this would also enhance the possibility to find factors that contribute to the prevention and treatment of dental anxiety/phobia [1].

This paper will describe a conceptual model for the treatment of dental anxiety and phobia, developed in collaboration between the Institute of Odontology, Sahlgrenska Academy, University of Gothenburg and the Public Dental Service, Region Västra Götaland, Sweden. The opening of a specialised clinic in the late 1970s involved a close relationship between research and the clinical management of dentally anxious patients [1]. Over the years, treatment programmes have been developed, based on the clinical research performed within the clinic as well as on results from other international research centres, adjusting and improving our efforts to implement evidence-based treatment models for dental anxiety and phobia [2,3]. In this paper, we will also present our views on the diagnosis of dental anxiety/phobia and related comorbidity conditions and on evidence-based treatments of dental anxiety/phobia.

## 2. Method

This state-of-the-art paper is based on the current knowledge of the (i) aetiology and (ii) epidemiology of dental anxiety/phobia among adult individuals as well as (iii) diagnostic criteria specifically concerning fear, anxiety, and phobias using relevant international classifications developed by the World Health Organization and the American Psychiatric Association. Furthermore, this paper in detail describes the (iv) conceptual treatment model at the Dental Fears Research and Treatment Center (DFRTC) at the Public Dental Service, Gothenburg, Sweden. This conceptual treatment model, described below, is based on extensive clinical research over the course of more than 40 years. Moreover, the literature presented in this paper includes up-to-date scientific publications evaluating the treatment of dental anxiety/phobia. The positions held by the authors include clinical psychologist, professor in psychology (UW), senior dental consultant, professor in dental public health, specialist in endodontics (MH), and they have 20 years and 35 years, respectively, of clinical and research experience of dental anxiety/phobia.

## 3. Dental Anxiety, Aetiology and Dental Public Health

**Figure 1:**
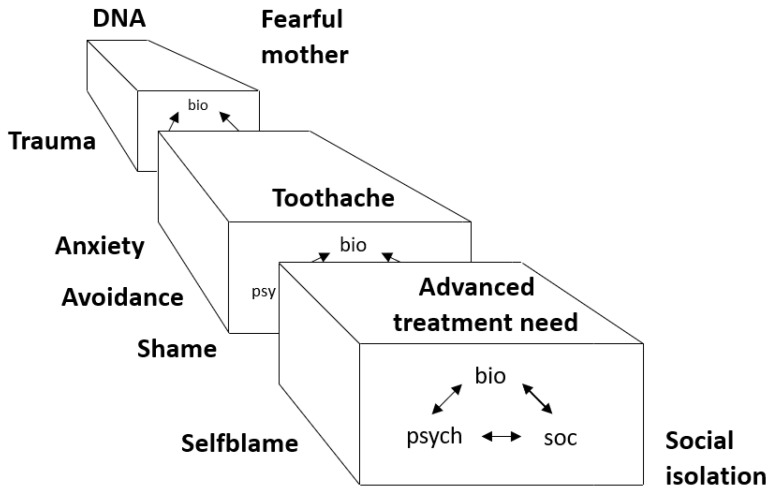
Intuitive analysis of the development of dental anxiety [1].

**Figure 2:**
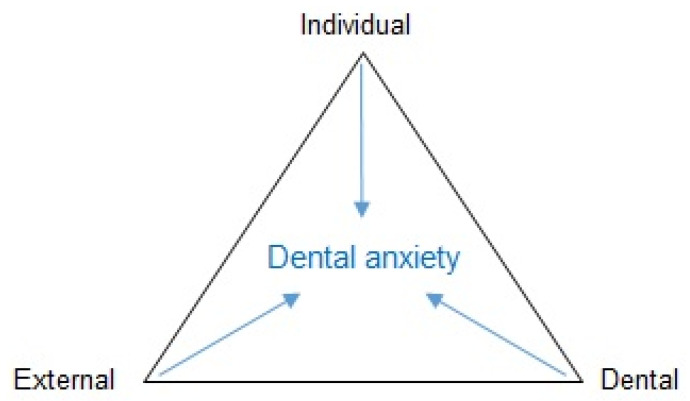
Aetiological model of dental anxiety [5].

However, first we wish to describe an up-to-date dental anxiety framework from a dental public health perspective, including the epidemiology of dental anxiety and the consequences and manifestations of dental anxiety. From an aetiological perspective, the development of dental anxiety and phobia may best be illustrated using a biopsychosocial model of the interaction of social, psychological, and biological factors over time (Figure 1) [1]. Like other anxieties and phobias, the start, or rather the initiation, of the condition (dental anxiety) implies a multifactor involvement, which is similar to many other diseases and illnesses. Thus, having a trauma or a fearful parent could initiate dental anxiety in childhood. However, for the maintenance of dental anxiety, other factors may be drivers, such as avoidance of dental care, social isolation, and advanced oral treatment needs (Figure 1). A commonly applied conceptual model for multifactor aetiological components is the causal pie model described by Rothman [4]. One way to understand the initial components of the model is shown in Figure 2, highlighting the individual, dental, and external factors that contribute to the development of dental anxiety, albeit in different proportions in an individual setting [5]. Individual factors may include other psychological disorders such as general anxiety, depressive symptoms, and post-traumatic stress disorder. Also, temperament and genetic vulnerability to anxiety have been reported to some degree as individual factors. External factors may be the social environment in which the person lives, concerning attitudes toward dental care, social position, and cultural circumstances. Dental factors are usually based on classical conditioning and direct learning. Negative and traumatic experiences in the dental setting are the main causes, including poor communication and behaviour from dental staff but also a strong negative experience of pain related to dental treatment. Importantly, from a causal and treatment perspective, it is mainly the dental factors that we can target and alter specifically to reduce the level of dental anxiety to more normal levels and improve the related dental behaviour towards better and regular dental visiting habits [6]. From a causal perspective, personal and external factors cannot be produced or prevented in the dental setting, whereas the dental factors are accessible for prevention and treatment of dental anxiety [6].

**Figure 3:**
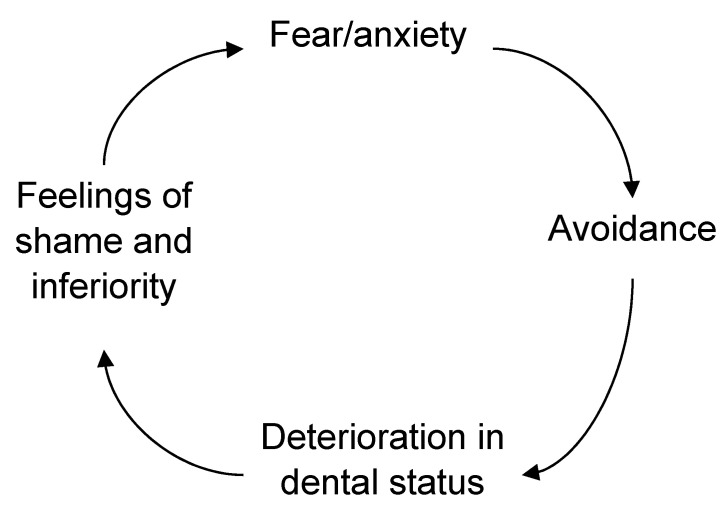
The vicious cycle of dental anxiety maintaining and enforcing dental anxiety [2].

However, one important aspect of the causal model in Figure 1 is the temporal relationship; namely, that risk factors change over time and that the causal factors that once initiated the dental anxiety are not necessarily the ones maintaining the dental anxiety or phobia later in life. Vicious cycles of dental anxiety have been presented in the literature, with the most widely acknowledged possibly being the one by Berggren [2]. The important feature of that model (Figure 3) is the presentation of the different components of dental anxiety in a circular mode, making it possible to test these factors/effects of dental anxiety empirically [7], while the drawback would be the longitudinal aspects of changes to the factors maintaining the dental anxiety. Moreover, one feature of the vicious cycle is the avoidance of dental treatment, which, at the time of launching the model, was related to the definition of a specific phobia. Today, according to the DSM 5 (Diagnostic and Statistical Manual of Mental Disorders), avoidance of the situation or object is not a necessary feature of the phobia but may be replaced by endurance under extreme distress [8]. In addition, the vicious cycle implies that avoidance of dental care may lead to poor oral health and feelings of shame and inferiority. These factors can be the drivers of the maintenance of high dental anxiety.

## 4. Dental Anxiety and Prevalence

How common is dental anxiety and phobia among adults? In a newly published thesis, Svensson [9] compiled a table of dental anxiety prevalence studies from different countries globally. The great majority of studies measured dental anxiety using an established psychometric test that contained several items with summated scores and usually a cutoff score to discriminate for high/severe dental anxiety. Another common way to measure dental anxiety has been to use a single, global question. Very few studies have applied diagnostic classification systems such as the DSM 5 or the ICD-10 (International Statistical Classification of Diseases and Related Health Problems) [8,10]. The former allows an interpretation of the distribution of dental anxiety on a continuous scale while the latter establishes the diagnosis dichotomously. It is also a question of the use of measurement method, as the purpose may be either clinical research for group level estimation of dental anxiety or for use in clinical practice on an individual level for clinical decision making. Dental anxiety and phobia should be considered a dental public challenge since around 5–15% of the adult population globally reports high/severe dental anxiety or phobia. Even if the prevalence appears to have declined over several decades, one in every ten adults has dental anxiety [11,12,13,14]. Studies have shown a clear and significant association between dental anxiety and concomitant problems, such as irregular dental visiting habits, poor oral health, and low oral health-related quality of life [15,16,17,18,19].

## 5. Setting a Diagnosis of Dental Anxiety/Phobia

Why is a diagnosis considered important? Firstly, establishing a diagnosis according to the DSM 5 or ICD-10 means that the individual has the disease/illness or condition, which must be followed by a treatment plan and subsequent treatment after a joint decision by the patient and the health professional. In the case of dental anxiety/phobia, the significance of having a diagnosis should not be underestimated, as it is obviously the equivalent of a somatic diagnosis and important to us in clinical practice. It may also be of importance when it is related to the cost of dental care and the prioritisation of different treatments and measures in health care as resources are generally not infinite. Moreover, establishing a diagnosis of dental anxiety/phobia may be the combined efforts of a dentist and a psychologist and constitutes the basis for a thorough anamnesis/examination, treatment plan, and treatment.

Specific phobia is defined in the DSM 5 [8] in summary as follows: Specific phobia is characterized by a marked and excessive fear or anxiety that consistently occurs upon exposure or anticipation of exposure to one or more specific objects or situations (e.g., proximity to certain animals, flying, heights, closed spaces, sight of blood or injury, receiving an injection) that is out of proportion to actual danger. The phobic objects or situations are avoided or else endured with intense fear or anxiety. Symptoms persist for at least several months and are sufficiently severe to result in significant distress or significant impairment in personal, family, social, educational, occupational, or other important areas of functioning. We believe it is of the utmost importance in clinical practice to acknowledge the use of these criteria for dental anxiety as a specific phobia since it matters to the patient and the dental team from a health service perspective.

## 6. Conceptual Treatment Model

Below follows a description of a conceptual treatment model of dental anxiety/phobia. The treatment model has been developed over many years, as mentioned above. It is also important to acknowledge and characterise the patients being referred to and treated at the Dental Fears Research and Treatment Center (DFRTC) in Gothenburg, Sweden. Since dental anxiety may be seen as a continuum, different treatment strategies should be applied. Thus, individuals with low and moderate dental anxiety may be managed through good communication by the staff, rapport building, enabling of patient control in the dental setting, and providing information about the dental care. Newton and co-authors have suggested different approaches to the management of dental anxiety, depending on the level of the dental anxiety [20]. They also added another dimension that is decisive for the choice of treatment based on the oral treatment need, i.e., urgent/not urgent treatment need. Thus, for low and moderate levels of dental anxiety, the above management strategy was suggested, and for high dental anxiety combined with no urgent treatment need, Cognitive Behavioural Therapy (CBT) (see below) was proposed. Finally, patients with high dental anxiety and an urgent oral treatment need should be treated with dental therapy under sedation, for example, with nitrous oxide, conscious sedation, or general anaesthesia but no CBT according to the authors. However, we argue that patients may very well be subjected to CBT after their acute dental need is resolved, and they agree to and are motivated for CBT.

CBT is the collective name for psychotherapies addressing psychological problems by helping patients learn new and more functional behaviours and cognitions. CBT has a solid theoretical and empirical base in behavioural, social learning and cognitive psychology, including both experimental and clinical research. CBT includes several different interventions, selected depending on the target psychological problem of the specific patient, as clarified with a functional behavioural analysis (corresponding to a diagnosis). CBT treatments are structured and performed in collaboration between the therapist and patient.

The evidence-based interdisciplinary dental anxiety/phobia treatment is delivered at the DFRTC at the clinic of oral medicine by a unit that includes psychologists, dentists, dental nurses, and dental hygienists. Patients attending and treated at the DFRTC have severe dental anxiety and poor oral health [21]; see Table 1. Patients may also have other mental health issues, such as general anxiety, depression, and post-traumatic stress disorder (PTSD) [22,23,24].

Initially, both the oral and psychological status of the patient is investigated by a dentist and a psychologist at the dental clinic. The primary task is to establish a working collaboration with the patient, while acknowledging and normalising the patient’s strong anxiety reactions to being at a dental clinic and providing empathy, understanding, and realistic hope. These patients are usually very ashamed of their situation, and in many cases, their confidence in dental personnel is damaged due to previous negative experiences. Following the anamnestic interviews and, if possible (but never proposed at the first visit), an adapted clinical examination, the dentist and the psychologist make a diagnosis based on the patient’s oral status and psychological status, with the focus on dental anxiety. Most patients have both severe dental anxiety, that is, dental phobia, and deteriorated oral status (Table 1) [25]. Therapy options are then discussed with the patient, and motivational techniques are used.

In this conceptual treatment model, the primary treatment goal is to treat and possibly cure the patient’s dental phobia, while dental care is integrated in the dental phobia treatment. All the patient’s dental treatment needs are not necessarily remedied at the DFRTC, as patients are usually referred to general dentistry to start attending to their dental care on a regular basis after completing treatment at the DFRTC [26]. This practice also allows the DFRTC to help new patients.

**Figure 4:**
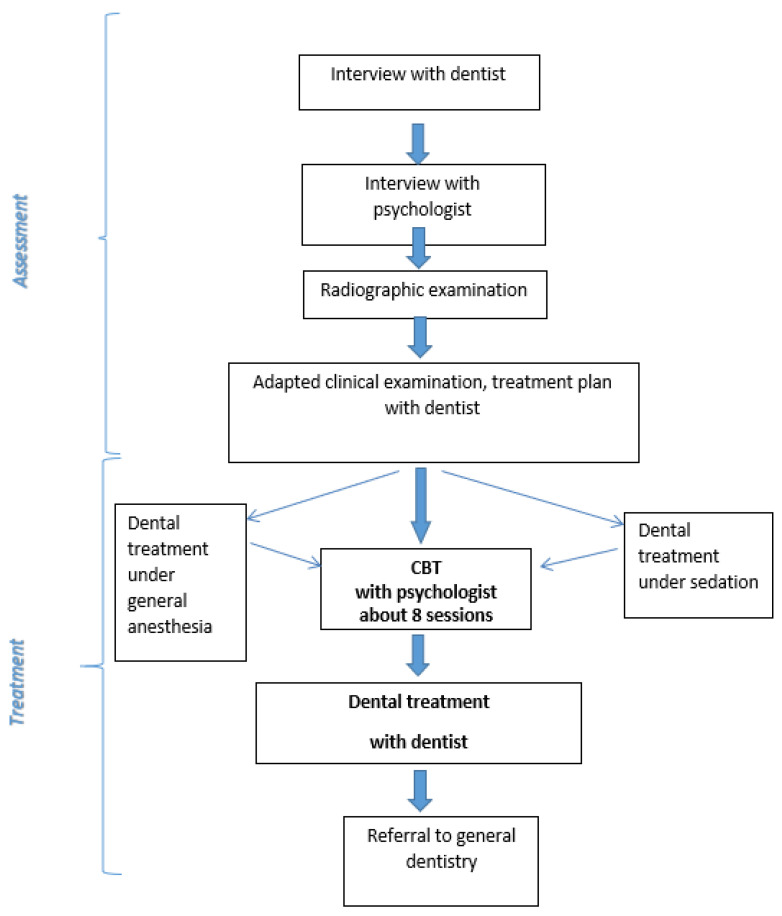
Flowchart for CBT treatment of dental anxiety/phobia [27].

**Figure 5:**
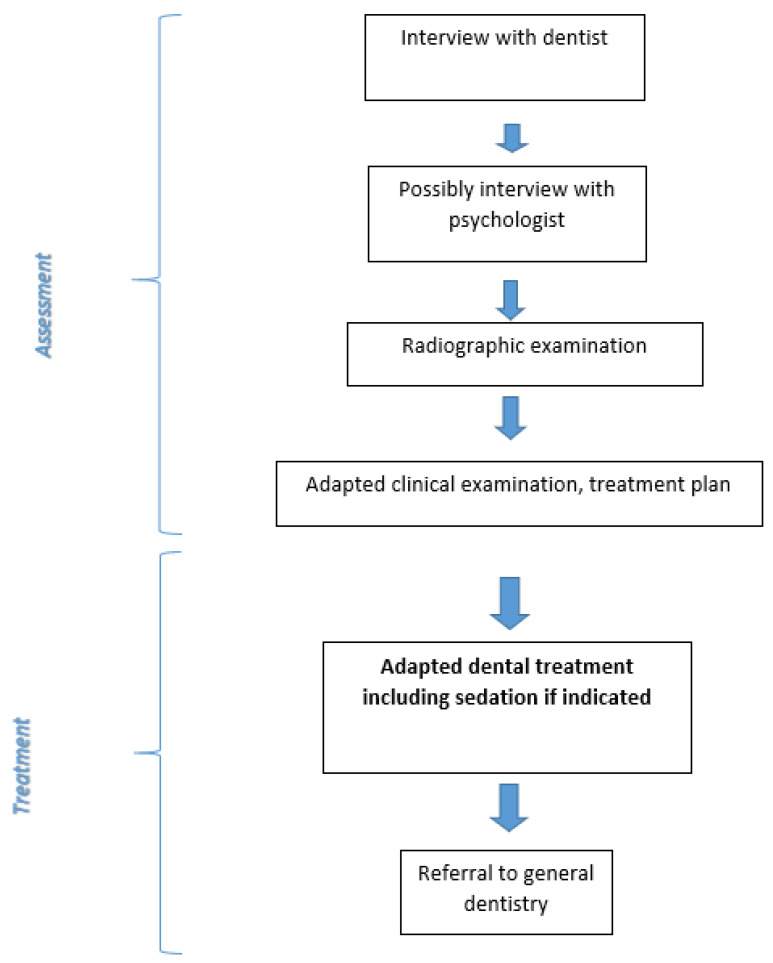
Flowchart for adapted dental treatment of dental anxiety/phobia [27].

Patients at the DFRTC are treated according to two different approaches: (i) dental phobia treatment with exposure-based multimodal cognitive behaviour therapy (CBT) [27] (Figure 4) or (ii) adapted dental treatment (Figure 5). The evidence-based treatment of specific phobias is CBT based on exposure (see below). Effective treatments for adults with dental phobia/severe dental anxiety using CBT in the dental setting are presented in the literature [28,29,30,31]. The second approach, adapted dental treatment, is recommended for patients with severe comorbidity (substance abuse, eating disorders, depression, general anxiety, or PTSD) or patients not motivated for CBT.

**Figure 6:**
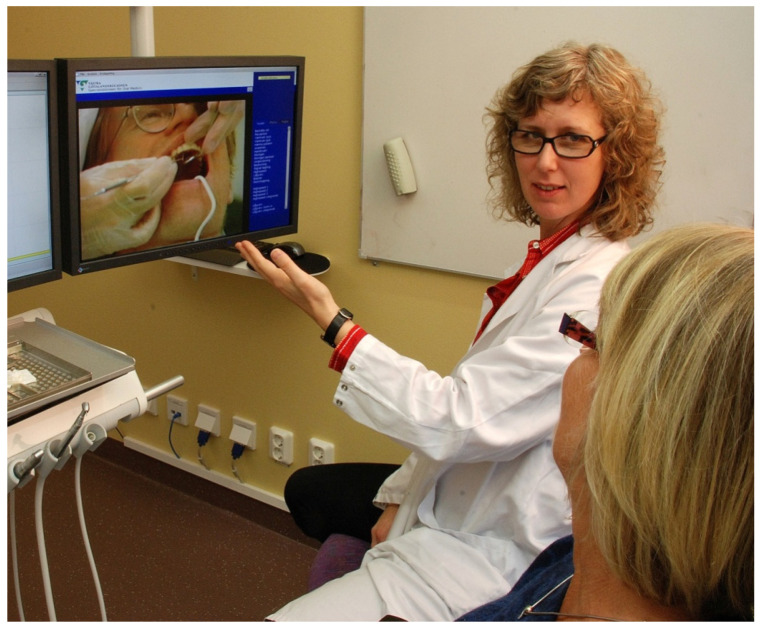
The CBT treatment setting, psychologist providing exposure in the dental operating room [34].

The CBT dental phobia treatment is delivered by a psychologist with formal CBT training (the same psychologist that performed the assessment) and includes a maximum of eight individual 50-min sessions held in a fully equipped dental operating room at the dental clinic (Figure 6). Several CBT interventions are included in the treatment manual, and the psychologist adapts the treatment to each specific patient following the case conceptualisation. However, the core interventions of exposure (gradual exposure to the anxiety-provoking situations and objects, while experiencing the anxiety reactions with support from the psychologist until the anxiety typically diminishes), applied relaxation (a technique for muscular relaxation including calm breathing), and cognitive restructuring (identification and challenging of dysfunctional thoughts and images) are always included. Likewise, also applied are behavioural functional analysis (corresponding to a diagnosis of the psychological problem), rationale (securing the patient’s understanding and consent to the therapy plan), and psychoeducation (teaching the patient about the nature of anxiety reactions), which are interventions included in all CBT treatments. Other commonly used interventions are self-assertiveness training (communication of personal needs and boundaries) and applied tension (a technique for muscular tension to hinder fainting due to a sudden drop in blood pressure). For a detailed presentation of the interventions and content of the treatment sessions, see Lundgren and Wide Boman [27].

The dental operating room serves as the basis for gradual exposure, where the most important part is the use of a battery of short film sequences of a non-dentally anxious patient undergoing a normal, conventional dental treatment, including situations where the patient in the film sequences interacts with the dentist and the dental nurse. The CBT patients see (and listen to) these films while sitting in the dental treatment chair, with the psychologist at their side assisting and coaching the patient. Exposure is a very demanding and anxiety-provoking intervention and must be delivered correctly to be effective (and not cause harm). Also, as many dentally anxious patients have traumatic experiences and possibly symptoms of post-traumatic stress disorder (PTSD), the psychologist adapts the exposure accordingly (using parts of the technique of prolonged exposure and handling strong emotional reactions).

As shown in Figure 4, initially, adapted dental treatment with sedation can be provided for patients with acute oral treatment needs before starting the CBT.

CBT treatment for dental phobia aims to increase the patient’s own competence and confidence to manage dental care on a regular basis within general dentistry and not be dependent on a specific person to manage the dental care. As shown in Figure 4, CBT with the psychologist is always followed by dental treatment with the same dentist who performed the initial assessment. The psychologist usually does not attend the dental treatment sessions; however, they are thoroughly planned by the psychologist together with the patient and can be seen, using modern CBT terminology, as “behavioural experiments” to test new behaviours, get encouraged by the dental team, and gain new experiences. At the same time, some of the patient’s oral treatment needs are met, which is rewarding and improves the patient’s oral health.

Finally, after at least two or possibly three or more dental treatment sessions, the patients usually report considerably lower levels of dental anxiety and improved ability to undergo dental treatment using the new skills in interactions with the dental team. The patient is then referred to general dental care within the Public Dental Service or private dental care, with instructions to the new dentist to facilitate further treatment [32,33].

Patients not suitable for or not at all motivated for CBT undergo adapted dental treatment (Figure 5) with the dental team. The assessment made by the psychologist can provide valuable information to the dental team concerning therapy planning and is an intervention that may help the patient better understand the situation. In adapted dental treatment, different forms of sedation can be used, and in addition to providing acute treatment, the focus is on exploring ways to facilitate further regular dental care. Patients in adapted dental care are usually referred to general dentistry after a treatment period, and the dentist then provides detailed instructions to the next dentist.

In Sweden, dental phobia treatment is mainly covered by the National Board of Health and Welfare since 1999, making it accessible in all regions in Sweden at a moderate cost for patients. However, to qualify for inclusion in this financial model, strict regulations apply regarding the phobia diagnosis, the competence of the care providers, and the interdisciplinary collaboration between dentists and psychologists (or therapists) during both the assessment and the treatment [34].

In 2017, Region Västra Götaland in Sweden decided on new regional clinical guidelines for adult individuals with severe dental anxiety/phobia, including the provision of specialised CBT administered by psychologists working in interdisciplinary collaboration with dental personnel at the same dental clinic to patients in the region. Systematic literature reviews, including meta-analyses, have clearly indicated CBT as the most effective treatment for adult individuals with severe dental anxiety [28,29,30,31]. These regional clinical guidelines improve the treatment for dental anxiety patients and make it available to all citizens in the region, thereby providing more equal evidence-based dental care.

In our experience, the CBT must be provided at a dental clinic for the following reasons: (i) for the psychologist to deliver evidence-based treatment (CBT with exposure), the setting must be a dental treatment room with access to anxiety-provoking stimuli and situations; (ii) to complete CBT, the patients need to participate in behavioural experiments, that is, to undergo dental treatment; (iii) most of our patients have severely deteriorated oral status and, therefore, the CBT must be planned and integrated with dental treatment in a sensible way.

## 7. Concluding Remarks

There are other models for dental anxiety treatment in other countries. In Norway, for instance, dentists specialised in dental anxiety provide the CBT for dental anxiety after undergoing CBT training. In the Netherlands, psychologists provide part of the CBT in more complex cases followed by exposure therapy by the specialised dentist. However, in these countries, psychologists take an active part in the assessment and treatment planning for each patient. Also, one-session interventions have been presented [35,36,37]. There are obviously many reasons for the variations in the forms of interventions to treat dental phobia: differences in how the dental service is organised and structured, in the availability of appropriately trained dentists and CBT psychologists/therapists, and differences regarding financing. In addition, there may be differences in patient groups, such as the level of dental anxiety, psychiatric comorbidity, complex problems, and differences in oral treatment needs. However, exposure-based CBT is the preferred treatment following a diagnosis of dental phobia in an evidence-based dental care perspective.

In our experience, psychologists are the preferred caregivers for treatment using CBT in patients with severe dental anxiety/phobia due to their expertise in theory-based psychological interventions and in the assessment and conceptual understanding of behaviour problems and mental distress from a psychological perspective. Dental anxiety is still a major problem affecting a substantial number of individuals and is related to impaired oral health and poor oral health-related quality of life [17,19]. Effective evidence-based treatments exist and should be provided in interdisciplinary collaboration within the dental setting to improve oral health and oral health-related quality of life in the population.

## Figures and Tables

**Table 1:** Characteristics of adult patients attending DFRTC ^#^ referred for dental anxiety [25]. Age, gender distribution, dental status (Decayed Missing Filled Teeth (DMFT)), dental anxiety (Dental Anxiety Scale (DAS), Dental Fear Survey (DFS)) and general anxiety (HAD-A) and depression (HAD-D) for the total group and a comparison between subjects without root remnants and with root remnants.

	Total Group	Root Remnants	
		No	Yes
	*n* = 148	*n* = 63	*n* = 85
Male/Female (%)	39/61	38/62	40/60
	mean (sd)	mean (sd)	mean (sd)
Age, years	36.1 (9.9)	33.7 (6.8)	38.0 (11.3) *
Decayed teeth	8.1 (5.2)	6.0 (4.4)	9.6 (5.2) **
Missing teeth	3.4 (4.0)	2.1 (2.9)	4.5 (4.3) **
Filled teeth	7.1 (4.8)	7.7 (4.9)	6.6 (4.7)
DMFT	18.6 (5.6)	15.7 (6.1)	20.7 (4.9) **
DAS	17.2 (2.6)	17.1 (2.5)	17.3 (2.7)
DFS	79.8 (12.5)	79.0 (11.9)	80.5 (13.0)
HAD-A	12.4 (5.0)	11.1 (4.8)	13.3 (4.9) **
HAD-D	7.1 (4.2)	5.8 (3.4)	8.0 (4.5) **

^#^ Dental Fears Research and Treatment Center, * *p* < 0.05, ** *p* < 0.01.

## Data Availability

This paper presents results from previous research. For information about data availability, see the referred studies.
